# Combination of Angiotensin (1-7) Agonists and Convalescent Plasma as a New Strategy to Overcome Angiotensin Converting Enzyme 2 (ACE2) Inhibition for the Treatment of COVID-19

**DOI:** 10.3389/fmed.2021.620990

**Published:** 2021-03-18

**Authors:** Hawraa Issa, Ali H. Eid, Bassam Berry, Vahideh Takhviji, Abbas Khosravi, Sarah Mantash, Rawan Nehme, Rawan Hallal, Hussein Karaki, Kawthar Dhayni, Wissam H. Faour, Firas Kobeissy, Ali Nehme, Kazem Zibara

**Affiliations:** ^1^PRASE and Biology Department, Faculty of Sciences - I, Lebanese University, Beirut, Lebanon; ^2^College of Public Health, Phoenicia University, Zahrani, Lebanon; ^3^Department of Basic Medical Sciences, College of Medicine, QU Health, Qatar University, Doha, Qatar; ^4^Biomedical and Pharmaceutical Research Unit, QU Health, Qatar University, Doha, Qatar; ^5^Institut Pasteur, Paris 6 University, Paris, France; ^6^Transfusion Research Center, High Institute for Research and Education in Transfusion, Tehran, Iran; ^7^EA7517, MP3CV, CURS, University of Picardie Jules Verne, Amiens, France; ^8^School of Medicine, Lebanese American University, Byblos, Lebanon; ^9^Department of Biochemistry and Molecular Genetics, Faculty of Medicine, American University of Beirut, Beirut, Lebanon; ^10^Department of Human Genetics, McGill University, Montreal, QC, Canada

**Keywords:** ACE2, SARS-CoV-2, COVID-19, lung pathology, cardiovascular pathology, convalescent plasma (CP), Angiotensin 1-7 (Ang1-7), combination therapy

## Abstract

Coronavirus disease-2019 (COVID-19) pandemic caused by severe acute respiratory syndrome coronavirus 2 (SARS-CoV-2) is currently the most concerning health problem worldwide. SARS-CoV-2 infects cells by binding to angiotensin-converting enzyme 2 (ACE2). It is believed that the differential response to SARS-CoV-2 is correlated with the differential expression of ACE2. Several reports proposed the use of ACE2 pharmacological inhibitors and ACE2 antibodies to block viral entry. However, ACE2 inhibition is associated with lung and cardiovascular pathology and would probably increase the pathogenesis of COVID-19. Therefore, utilizing ACE2 soluble analogs to block viral entry while rescuing ACE2 activity has been proposed. Despite their protective effects, such analogs can form a circulating reservoir of the virus, thus accelerating its spread in the body. Levels of ACE2 are reduced following viral infection, possibly due to increased viral entry and lysis of ACE2 positive cells. Downregulation of ACE2/Ang (1-7) axis is associated with Ang II upregulation. Of note, while Ang (1-7) exerts protective effects on the lung and cardiovasculature, Ang II elicits pro-inflammatory and pro-fibrotic detrimental effects by binding to the angiotensin type 1 receptor (AT1R). Indeed, AT1R blockers (ARBs) can alleviate the harmful effects associated with Ang II upregulation while increasing ACE2 expression and thus the risk of viral infection. Therefore, Ang (1-7) agonists seem to be a better treatment option. Another approach is the transfusion of convalescent plasma from recovered patients with deteriorated symptoms. Indeed, this appears to be promising due to the neutralizing capacity of anti-COVID-19 antibodies. In light of these considerations, we encourage the adoption of Ang (1-7) agonists and convalescent plasma conjugated therapy for the treatment of COVID-19 patients. This therapeutic regimen is expected to be a safer choice since it possesses the proven ability to neutralize the virus while ensuring lung and cardiovascular protection through modulation of the inflammatory response.

## Introduction

Starting November 2019, several cases of pneumonia of unknown etiology were reported in Wuhan, China (1). The causal agent was identified as severe acute respiratory syndrome coronavirus-2 (SARS-CoV-2). The newly identified betacoronavirus differs from severe acute respiratory syndrome coronavirus (SARS-CoV-1) and Middle East respiratory syndrome coronavirus (MERS-CoV); however, it causes similar symptoms associated with pneumonia (2–4). In contrast to SARS-CoV-1, which caused the 2002 outbreak, SARS-CoV-2 exhibits a higher risk of transmission as evident from the rapid global rise in the number of Coronavirus disease 2019 (COVID-19) cases (2–4). As of mid February 2021, more than 110 million cases have been confirmed, and nearly 2,400,000 deaths were reported globally, with the rapid increase of numbers in many countries.

SARS-CoV-2 is mainly transmitted from person to person through respiratory droplets, contact, aerosol, or oral-fecal transmission (5, 6). While most reported COVID-19 cases present mild to moderate pathology, 20% of infected patients may develop severe disease and need intensive care (7–12). Severe cases progress to acute respiratory distress syndrome (ARDS) after 8–9 days of symptoms onset (1). ARDS seen in severe COVID-19 cases is characterized by difficulty in breathing and low blood oxygen level, leading to respiratory failure, which is the main cause of death in 70% of fatal COVID-19 cases (8–10). Plasma analysis of severe cases showed a massive release of cytokines by the immune system (cytokine storm) in response to the viral infection and/or potential secondary bacterial and fungal infections (8, 9). This uncontrolled inflammation induced by SARS-CoV-2 infection results in multi-organ damage, leading to organ failure (10).

Certain groups of the population are more susceptible to SARS-CoV-2 infection (11–14). The case fatality rate (CFR) seems to be age-dependent, with a higher percentage in the elderly, especially men. SARS-CoV-2 may have a higher transmissibility than SARS-CoV-1 and MERS-CoV (1, 8, 15, 16). Patients with pre-existing comorbidities such as chronic obstructive pulmonary disease (COPD), cardiovascular diseases, hypertension and type 2 diabetes mellitus, are more likely to display a severe course and to have higher mortality rates (13–18).

Initial research on SARS-CoV-2 demonstrated that it binds to host cells using the angiotensin-converting enzyme 2 (ACE2), similar to SARS-CoV-1. SARS-CoV-2 binds to ACE2 proteins as a receptor in bats, civet cats, swine, cats, ferrets, non-human primates, and humans (16–18).

The binding affinity of SARS-CoV-2 RBD to ACE2 seems stronger than that of SARS-CoV-1. Besides, SARS-CoV-2 has evolved to use a wide array of host proteases (transmembrane protease serine 2 (TMPRSS2), cathepsin L/B, furin, trypsin, etc.) for S-protein priming, thus facilitating cell entry following receptor binding. This may explain the considerably larger global influence of COVID-19 than the initial SARS epidemic of 2003 (19–21). It is worth noting that new SARS-CoV-2 strains, namely the British (B.1.1.7) and South African variants (B.1.351), that emerged toward the end of 2020 show increased transmission capacity, associated with increased interaction force between Spike and ACE2 proteins due to viral mutations (22, 23). In addition, the RBD/ACE2 mediated SARS-CoV-2 entry into cells is followed by subsequent downregulation of surface ACE2 expression (24). Several reports indicate that the reduction in ACE2 function influences blood pressure, perturbs fluid/electrolyte balance, enhances inflammation, and vascular permeability in the airways, and facilitates the development of multiorgan damage from SARS-CoV-2 infections (25–29). Consequently, ACE2 appears as a critical factor in understanding COVID-19 pathology and a potential target for COVID-19 treatment.

## ACE2 is Part of a Complex System

**Figure 1:**
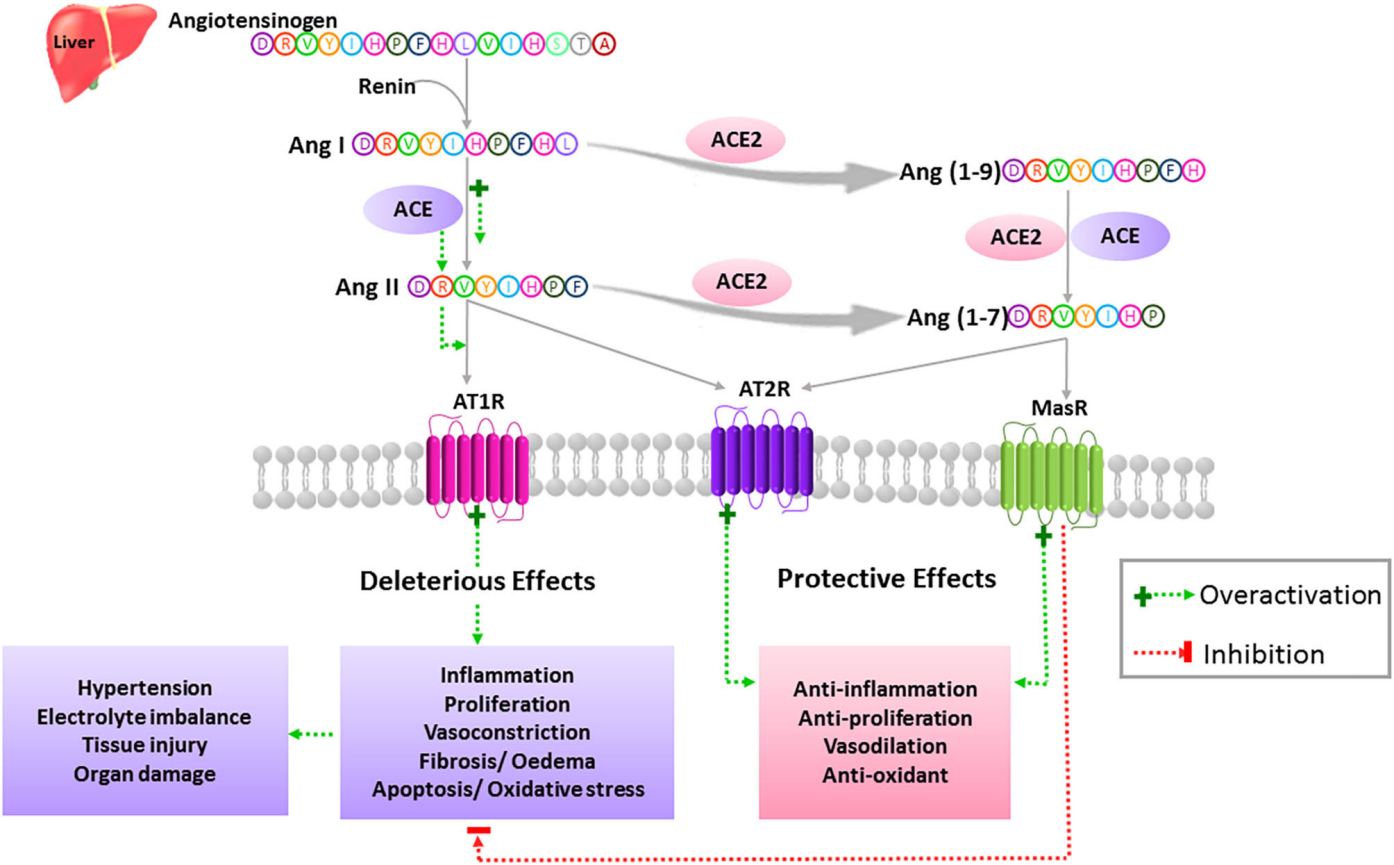
Simplified view of the extended RAS. In the classically described RAS, the inactive zymogen angiotensinogen secreted mainly by the liver, is converted into Ang I by the action of the renal aspartyl protease, renin. Ang I is then cleaved by ACE to generate the Ang II octapeptide. Ang II is a multifunctional hormone that regulates blood pressure and fluid homeostasis. This peptide exerts its actions through binding to two main receptors, AT1R and AT2R, which are typical seven transmembrane GPCRs. More specifically, Ang II mediates its vasoconstrictor effects by stimulating AT1Rs while AT2Rs are known to balance the actions of AT1Rs via activation of vasodilatory pathways. Dysregulation of the RAS in favor of the ACE/Ang II/AT1R axis leads to the pathogenesis of hypertension as well as tissue injury and multi-organ damage through activation of oxidative stress, proliferation, inflammation, fibrosis, edema, and apoptosis. Ang II can either bind to its receptors or is further cleaved to yield degradation products such as Ang (1-7). This bioactive peptide is produced mainly by means of ACE2. Ang (1-7) is obtained directly by the action of ACE2 on Ang II or indirectly by generating Ang (1-9) as an intermediate product. Ang (1-7) exerts its protective effects through activation of the AT2R and MasR and opposes the described detrimental effects of the ACE/Ang II/AT1R axis.

ACE2 is a membrane-bound glycoprotein of 805 amino acids that exhibits 40% identity and 61% similarity to human angiotensin converting enzyme (ACE). Full-length ACE2 consists of a heavily glycosylated N-terminal signal sequence containing the active site, a hydrophobic transmembrane sequence, and a short C-terminal cytoplasmic tail. A soluble and catalytically active form of ACE2 can be also produced by several mechanisms, including the action of ADAMs family members of zinc metalloproteases (30–33). ACE2 is the main enzyme involved in the production of the Ang (1-7) peptide of the renin-angiotensin system (RAS) (Figure 1). The latter comprises successive enzymatic reactions that regulate multiple biological processes, including cellular growth, proliferation, migration, extracellular matrix remodeling, and inflammation. While RAS includes multiple enzymatic axes leading to the production of different bioactive peptides, local tissue effects of RAS are driven mostly by the balance between the pro-inflammatory/pro-fibrotic and anti-inflammatory/anti-fibrotic actions of Ang-II and Ang (1–7), respectively (34, 35) (Figure 1).

Ang II is produced by the cleavage of Ang I by the angiotensin-converting enzyme (ACE), and it exerts its pro-inflammatory/pro-fibrotic effects by binding to its angiotensin type 1 receptor (AT1R) (Figure 1). On the other hand, ACE2 is the most potent Ang (1-7) generating enzyme. By its single catalytic domain, ACE2 can produce Ang (1-7) directly by cleaving one amino acid from the C-terminal domain of Ang II or indirectly through two successive cleavage reactions from Ang I (36) (Figure 1). Of note, Ang II is the preferred substrate for ACE2, with an affinity of 400-fold higher than that of Ang I (37). Hence, Ang-(1–7) production is based on both ACE2 levels and the ACE/ACE2 ratio.

Ang (1-7) exerts its effects by binding to and activating the G-coupled receptor Mas (MasR), which opposes the pro-inflammatory and pro-fibrotic actions of Ang II through AT1R activation (38, 39). Moreover, MasR can inhibit Ang II-mediated actions by hetero-oligomerization and inhibition of AT1R. Ang (1-7) can also exert its protective effects by binding directly to the angiotensin type 2 receptor (AT2R) (40, 41). Interestingly, the AT2R can exert similar protective effects when bound by Ang II (34, 35). Thus, the crucial role of ACE2 in RAS stems from the fact that it cleaves and opposes the action of Ang II (Figure 1). Consequently, it has a beneficial role in many diseases such as diabetes and cardiovascular diseases (CVD) (33), as well as in COVID-19 (42).

## Role of the ACE2/ANG (1-7)/MasR Axis in Pulmonary Physiology and Pathology

RAS gained an increased complexity and appreciation with the identification of local RAS in different tissues, including the brain, kidneys, heart, ovary, pancreas, and the vascular wall, independent of the well-known traditional circulatory RAS. Also, the discovery of additional RAS components (alternative enzymes, receptors, and bioactive angiotensin peptides) has extended the system's role far beyond blood pressure regulation and body electrolyte balance. Indeed, novel actions for each member of the RAS are continuously discovered in physiology and diseases (43).

ACE2 is expressed in human airway epithelia and lung parenchyma, suggesting a role in the regulation of pulmonary physiology (44, 45). A large body of evidence has shown the protective role of the ACE2/Ang (1-7)/MasR axis in several models of lung injury, including SARS-CoV-1 mediated injury (25, 46). In fact, to protect from severe lung failure, ACE2 is known to inhibit Ang II production, ACE activity, and AT1R activation. Of note, the anti-inflammatory and anti-fibrotic responses of ACE2 are mediated by the production of Ang (1-7) bioactive peptide, which protects against acute lung failure via activation of MaSR and AT2R. More specifically, Ang (1-7)/MasR exerts its beneficial effects by inhibiting ERK1/2 and NF-κB signaling pathways in a rat model of ARDS (47) and a mouse model of chronic allergic lung inflammation (44, 48). Magalhaes et al. also showed that MasR knockout mice failed to attenuate inflammation and pathological lung remodeling and presented aggravated asthma due to disruption of the protective arm of the RAS (49). The same research group confirmed that Ang (1-7) infusion resolved inflammation through correction of eosinophile defective apoptosis leading to lung damage (48). Ang (1-7) drug was also demonstrated to prevent bronchial responsiveness, a hallmark sign of chronic asthma (44).

Dysregulation of ACE/ACE2 balance leads to impaired lung function due to inflammation, fibrosis, and lung edema. The latter phenomenon is most probably induced via increasing pulmonary blood vessels' permeability (25, 46, 47). Elevated ACE concentrations have been detected in many potentially fibrotic lung diseases, including idiopathic pulmonary fibrosis (50) and ARDS (51, 52). Similar pro-fibrotic effects were also observed in a mouse model of ARDS using the MasR antagonist A779 (53). Moreover, Ang II has been shown to stimulate lung fibroblast proliferation and procollagen production by stimulating AT1R and the autocrine action of TGFβ. Of interest, *losartan* (AT1R blocker) and *ramipril* (ACE inhibitor), and Ang (1-7) were shown to reduce lung collagen deposition in the same study (53, 54). Thus, the protective effects of ACE2 on the lungs can be attributed to the inactivation of the ACE/Ang II/AT1R axis in favor of the ACE2/Ang (1-7)/MasR-AT2R axis (25).

## Role of ACE2 in the Pathology of COVID-19

The expression of ACE2 in human airways and lung tissues highlights its role in respiratory infections, including SARS-CoV-1 and the related human respiratory coronavirus NL63 (55). Although ACE2 is the main door for virus entry, the total ACE2 activity seems to be protective. In fact, several reports mentioned that ACE2 could be downregulated after virus entry and/or host cell lysis, as in SARS-CoV-1. The latter is reported to reduce ACE2 expression at the cell surface as well as the release of active ACE2 ectodomains (56, 57). This fact may further accentuate the pathogenesis of COVID-19, as ACE2 is shown to be protective in several models of lung injury, including SARS-CoV-1 mediated injury (25, 46).

Both SARS-CoV-1 and SARS-CoV-2 use the same receptor, ACE2, to infect cells. Interestingly, SARS-CoV-2 was shown to have a higher affinity for ACE2 than SARS-CoV-1 (58–60). Higher affinity values could be related to the dynamic of infection and the rapid spread that characterize this virus (61). For instance, mutations that increase the infectivity on RBD could explain why SARS-CoV-2 is more infectious than SARS-CoV-1 (62). Notably, mutations affecting SARS-CoV-2 have also been reported. In fact, by the end of August 2020, the C.1 lineage of SARS-CoV-2 presenting one amino acid substitution, D614G, on the spike protein, among 16 other nucleotide mutations, became the predominant lineage in South Africa (63). Analyses of over 28,000 SARS-CoV-2 spike protein gene sequences revealed that the D614G amino acid substitution facilitates the binding to ACE2 receptor and thus enhances viral replication in human lung epithelial cells and primary human airway tissues. This might account for its increased virulence to the respiratory system (64, 65). In addition, the 501Y.V2 variant that appeared in South Africa in December 2020 showed three important mutations in RBD (K417N, E484K, and N501Y) that are most probably correlated with functional significance (66). Another study on the B.1.1.7 British lineage revealed that the N501Y mutation of the SARS-CoV-2 spike protein is linked with increased interaction with ACE2 receptor, which explains its high infectivity rate (23).

**Figure 2:**
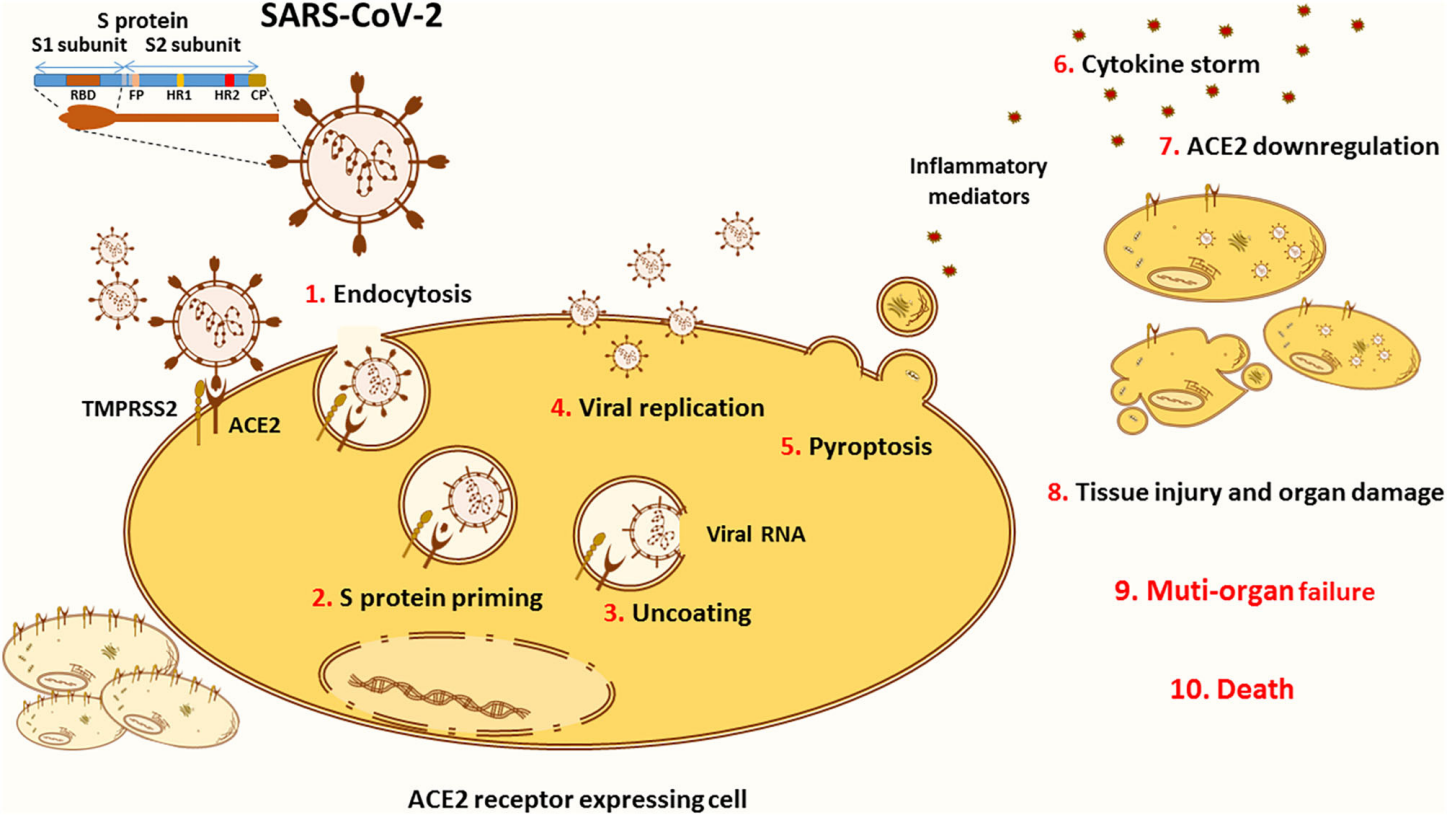
ACE2 in the pathology of COVID-19. The novel SARS-CoV-2 infects the cells through binding to its main receptor ACE2. The latter recognizes the RBD of the S1 subunit and allows the endocytosis of the virus (1). Once exposed to the action of proteases, such as the cellular TMPRSS2, the S1 subunit is cleaved away to ensure S protein priming (2). The fusion peptide (FP) of the S2 subunit is thus exposed to the cellular membrane. The FP initiates the fusion of the viral coat to the endosomal membrane enabling the uncoating of the virus (3). Released into the cytoplasm of the host cell, the viral RNA hijacks the cellular machinery to produce novel viral particles (4). Massive viral replication is thought to be linked with pyroptosis (5), an inflammatory form of apoptosis associated with the release of inflammatory mediators that activate various immune cells in order to create a cytokine storm (6) contributing to the pathogenesis of COVID-19. Viral entry and cellular apoptosis lead to ACE2 downregulation (7), thus stimulating the harmful effects of the ACE/Ang II/AT1R axis. Altogether, these processes are translated into tissue injury and multi-organ damage (8) that can evolve into respiratory, cardiac, hepatic, and/or renal failure (9), causing death (10).

The SARS-CoV-2 entry into target cells is initiated by the binding of the surface unit, S1, of the spike (S) protein to the ACE2 cellular receptor (Figure 2). The entry then requires S protein priming by TMPRSS2 serine proteases, which entails S protein cleavage and allows the fusion of viral and cellular membranes (67). Of note, several studies highlighted TMPRSS2 implication as a critical factor for the spread of clinically relevant viruses, including influenza A and other coronaviruses (68–70). One study conducted on a cohort of Italian patients announced that COVID-19 susceptibility is determined by genetic variability of TMPRSS2 known to be involved in SARS-CoV-2 entry into target cells. In this context, the data showed that in comparison to other European populations, Italians might have a higher level of TMPRSS2 or activity since they show a significant decrease in the deleterious variants of this protein. This can be considered as a risk factor for a more severe illness course (71). TMPRSS2 mediated activation of S protein priming enables viral infection of ACE2 positive cells. This initial phase is associated with viral replication and leads to pyroptosis, an inflammatory form of apoptosis, inducing lung injury. Of note, the formation of new viruses is also correlated with the induction of a cytokine storm via activation of various immune cells (56, 72).

## Differential Response to COVID-19 Could be Related to ACE2 Expression

Severe COVID-19 cases are mostly observed among elderly patients, with males suffering from chronic comorbidities such as cardiovascular and cerebrovascular diseases, diabetes, and others. Recently, it was established that risk factors including age, male sex, and hypertension provide a convenient tool to identify high-risk individuals. Hypertension elucidates the involvement of RAS in the pathogenesis of COVID-19 due to the interplay between SARS-CoV-2 and ACE2 (73–75). It is believed that ACE2 expression pattern in different organs, tissues, and cell types is permissive for the susceptibility of SARS-CoV-2 infection since ACE2 receptor permits coronavirus entry, replication, spread, and pathogenesis (76).

In order to identify the initial reservoirs of SARS-CoV-2, ACE2 expression levels were assessed within the lung and the upper/lower airway epithelium. ACE2 was expressed at low levels in the respiratory tract, and it was expressed in multiple epithelial cell types across the lower airway, with the highest expression being observed in club epithelial cells in comparison with basal and ciliated epithelial cells (77). ACE2 expression was also detected in alveolar type II cells in the lung parenchyma (77). In fact, alveolar type II cells, which account for only 5% of the alveoli, are essential to maintain lung elasticity and act as progenitors for alveolar type I cells responsible for gas exchange. Thus, SARS-CoV-2 might be responsible for depletion of the alveolar stem cells leading to the development of irreversible lung injury (56, 76). Remarkably, in the upper airway, nasal epithelial cells, including goblet and ciliated cells, showed the highest expression of ACE2 among all investigated cells in the respiratory tree; thus, highlighting their role in facilitating initial viral entry, transmission, and clearance (45, 77). Moreover, ACE2 expression is dynamic and depends on the differentiation status of epithelial cells. For instance, it is worth noting that differentiated epithelial cells expressing a higher level of ACE2 are readily infected in comparison to undifferentiated cells with low ACE2 expression (55). These findings may raise the theoretical assumption that the differential response to COVID-19 in patients could be in some aspects attributed to changes in ACE2 expression.

In this context, it has been reported that long term smokers express high levels of ACE2 receptor (78), namely in type-2 pneumocytes and alveolar macrophages (79), making them at high risk of infection by SARS-CoV-2 (78). This elevated expression occurs through activation of the α7 subtype of the nicotine acetylcholine receptor (α7-nAChR) (80). This is further highlighted in a recent meta-analysis revealing that patients with a history of smoking, as well as active smokers, recorded a significant severity of COVID-19 (81). In fact, cigarette smoking promotes alterations in the respiratory tract that might increase the risk of viral infections through multiple mechanisms such as impairment of mucociliary clearance, mucus hypersecretion, fibrosis, and dysfunction of the epithelial barrier. These mechanisms are accompanied by alterations in the immune response, eventually harming the function of the lungs, including gas exchange (82–86).

Although different studies have reported the upregulation of ACE2 expression in the lungs of cigarette smokers (78, 87), surprisingly, a very recent study revealed a decrease in the levels of ACE2 receptor in both alveolar and bronchial epithelial cells of mice exposed to cigarette smoking. Additionally, an *in vitro* study on Calu3 human lung cancer cell line treated with cigarette smoke showed no effect on ACE2 levels but effectively inhibited SARS-CoV-2 replication (88). These conflicting results urge for more work to clarify the role of cigarette smoking on ACE2 expression, SARS-CoV-2 infection and its severe respiratory complications.

## Differential Response to COVID-19 and ACE2 Expression in Cardiovascular Patients

RAS dysregulation, highlighted by Ang II upregulation, is associated with the pathogenesis of CVD. RAS inhibitors, such as ACE inhibitors (ACEI) and AT1R blockers (ARBs), are commonly used for the treatment of CVD patients. ACEI are known to downregulate the expression of Ang II, whereas ARBs are known to block Ang II-mediated detrimental effects (41). Due to the beneficial effects for the activation of the ACE2/Ang (1-7) axis, there has been substantial interest in considering the effect of RAS inhibitors on ACE2 expression in patients with CVD. Previous evidence in several animal models indicated that certain ARBs and ACEIs exhibit the ability to increase ACE2 mRNA and protein expression levels in the heart (89, 90). More importantly, ARBs were shown to alter the expression of ACE2 more consistently than ACEI (91). Although some animal studies displayed an elevation in ACE2 expression under the effect of RAS inhibitors (92), other studies did not. In this context, it has been reported that ACEI did not alter the activity of ACE2 *in vitro* (93). In another study, the use of the ACEI *ramipril* or of the ARB *valsartan* did not increase cardiac ACE2 expression in a rat model of myocardial infarction (94).

In contrast to animal models, limited investigation has been conducted in humans to consider the influence of RAS inhibition on the expression of ACE2 (95). In fact, a human study involving patients with hypertension showed higher urinary ACE2 levels in patients treated with the ARB *olmesartan*, but not with other ARBs or the ACEI *enalapril* (96). Importantly, ACE2 upregulation has been mostly noticed in renal vasculature and in cardiac tissue. However, the outcomes differed depending on the RAS inhibitors used (97). Furthermore, most human studies relied on measuring the soluble ACE2 levels in the blood. It's worth noting that measuring the membrane-bound ACE2 expression *in vivo* is technically challenging. In this regard, an increase in soluble ACE2 levels may refer to a decrease in the membrane-bound form of ACE2. Therefore, the distinction between soluble and membrane-bound ACE2 must be clear (97).

Overall, upregulation of ACE2 expression in CVD patients under ARBs or ACEIs raised several theoretical assumptions that these treatment regimens might put them at a greater risk of infection by SARS-CoV-2 (60, 95).

## Possible Scenarios on Using ACE2-Based Treatments for COVID-19

**Figure 3:**
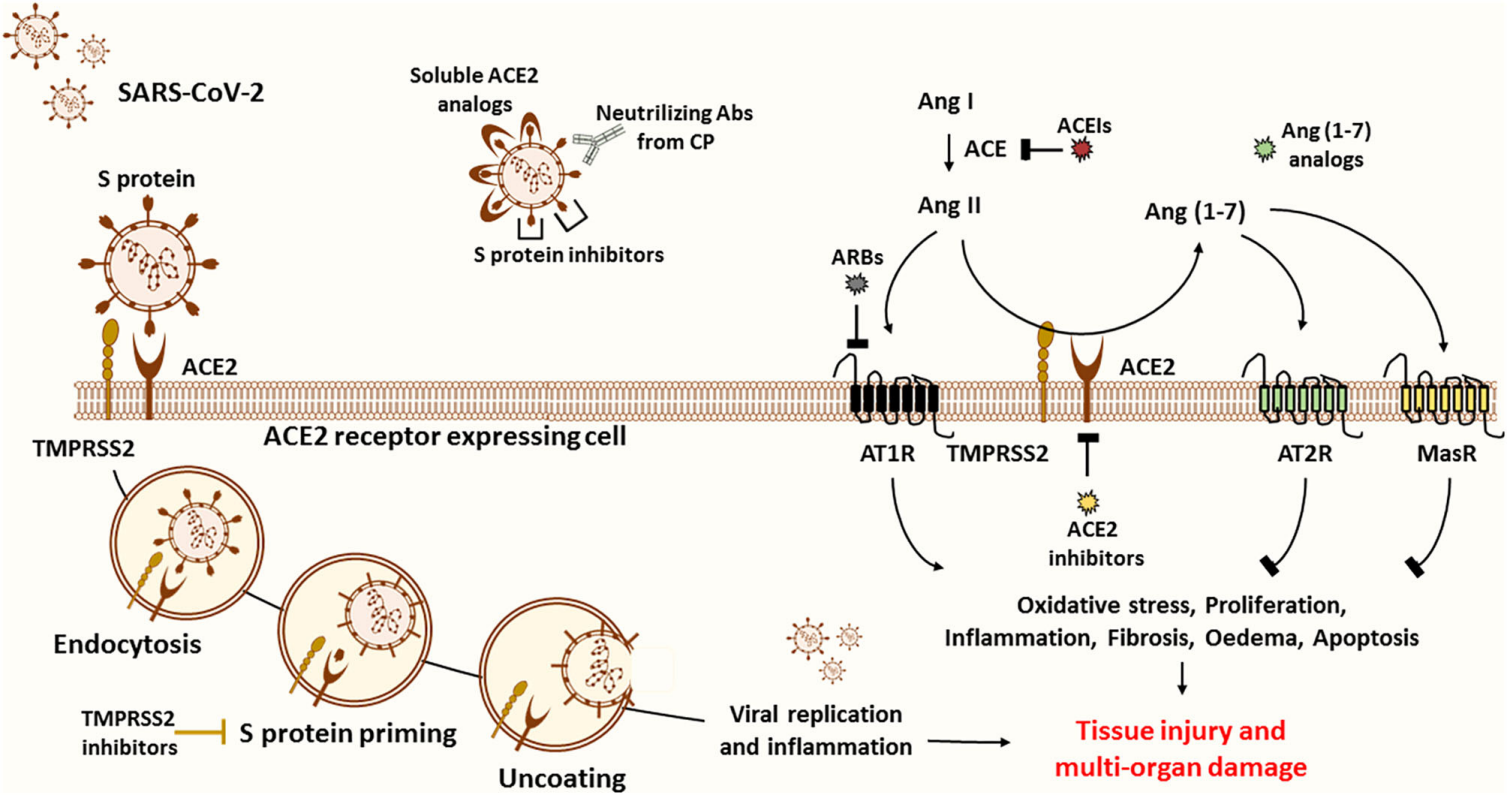
Possible treatment strategies for COVID-19. ACE2 is believed to be the main entry door for the SARS-CoV-2. ACE2 interaction with the RBD of the S1 subunit mediates viral entry into the host cell. To inhibit viral entry, researchers suggest the use of several drugs, including ACE2 inhibitors, soluble ACE2 analogs, S protein inhibitors, and transfusion of convalescent plasma from recovered patients. First, ACE2 inhibitors (pharmacological inhibitors and Abs) are more harmful than protective since ACE2 is known to be the primary source of the anti-inflammatory Ang (1-7) peptide. ACE2 inhibition and upregulation of Ang II expression stimulate the pathogenesis of many diseases through activation of AT1R. The latter stimulates oxidative stress, proliferation, inflammation, fibrosis, edema, and apoptosis, thus leading to tissue injury and multi-organ damage. Of note, Ang (1-7) counteract the ACE/Ang II/AT1R axis by activating the MasR and the AT2R. Second, soluble ACE2 analogs act as a trap competitively binding the virus to prevent cellular entry while rescuing ACE2 activity. Despite their beneficial effects, they can form a circulating reservoir of the virus. Third, spike protein inhibitors appear to be more promising to reduce disease severity. Fourth, another effective plan is based on the use of COVID-19 Abs from CP of recovered patients to neutralize the virus. This alternative has been proved to be safe and efficient in critically ill patients. Fifth, other therapeutic approaches encourage targeting S protein priming by means of protease inhibitors such as TMPRSS2 inhibitors to prevent the release of viral RNA into the cytoplasm of host cells, thus blocking subsequent viral replication and inflammation. In fact, virus entry and apoptosis are associated with ACE2 downregulation and consequently Ang II overproduction. Sixth, recent reports propose the use of Ang (1-7) analogs to block excessive inflammation through stimulation of the protective arm of the RAS. Importantly, Ang (1-7) drug formulation is useful in the management of several diseases, including cancer. Seventh, others suggest that ARBs and ACEIs might be useful in blunting the detrimental effects of ACE/Ang II/AT1R axis. Of note, these could also upregulate the expression of ACE2 and thus the risk of viral entry. Based on the above, we encourage the adoption of CP and Ang (1-7) conjugated therapy to neutralize the virus while controlling the inflammatory process to ensure organ protection.

ACE2 appears as a potential target for COVID-19 treatment based on the fact that it is an entry receptor critically involved in mediating SARS-CoV-2 infection and on its central role in cardiac pathology as well as in lung damage (98). Some reports suggested introducing ACE2 blockers, such as the MLN-4760 chemical inhibitor, or targeted antibodies to disrupt the viral entry into cells (99, 100). This approach could be detrimental to the risk of reducing ACE2 protective and anti-inflammatory activity, which further increases the susceptibility of lungs for more damage. Instead, viral entry could be impaired by protease inhibitors targeting TMPRSS2 protease implicated in SARS-CoV-2 cell entry, without risking the endogenous ACE2 activity (98) (Figure 3).

A better alternative to attenuate the viral load and infection, in comparison with ACE2 antibodies and pharmacological inhibitors, would be to deliver excessive soluble viral receptor analogs in order to intercept the viral binding to the membrane-bound ACE2. The conceptual principle is that soluble ACE2 may act as a trap, competitively binding and neutralizing the virus while rescuing cellular ACE2 activity and protecting the lungs from injury (99). In fact, a fusion protein of recombinant human ACE2 (rhACE2) was reported to show high affinity to SARS-CoV-2 binding domain and to neutralize the virus *in vitro* (101). Furthermore, Monteil et al. recently demonstrated that a clinical-grade rhACE2 is capable of reducing the viral load of SARS-CoV-2 infection in Vero-E6 cells and of blocking its entry into the cells. This study revealed that rhACE2 could inhibit SARS-CoV-2 infections in human organoids, such as the kidneys, during early stages of viral infection (102). Moreover, a clinical pilot study was planned (NCT number: NCT04287686) to deliver soluble rhACE2 infusions in a small COVID-19 patient cohort in China. Nevertheless, this study was withdrawn for non-stated reasons. Regardless, a large phase II clinical trial has been initiated at the beginning of April by the Austrian pharmaceutical company APEIRON to treat COVID-19 patients with APN01-rhACE2, in Austria, Germany, and Denmark (98). On the other hand, soluble ACE2 appears to have a short half-life and may lack the capacity to overcome massive virus infection. This agent could form a reservoir of circulating viruses, thus increasing its propagation (103–105) (Figure 3).

Altogether, clinical trials using ACE2-based treatments are eagerly awaited to exclude possible adverse effects and to prove their promising potential to enhance the positive outcomes in patients infected with COVID-19.

## ARBs to Reduce the Side Effects of ACE2 Inhibition by COVID-19

It is believed that SARS-CoV-2 infection may cause an increase in lung injury and severe acute respiratory failure due to ACE2/Ang (1-7) downregulation. In addition, since Ang II is the major substrate of ACE2, inhibition of ACE2 following viral entry is generally associated with Ang II upregulation as well as activation of the AT1R. In fact, elevated levels of Ang II were reported in the plasma of SARS-CoV-2 infected patients, which were positively correlated with viral load and lung injury. Thus, the harmful effects of COVID-19 could be achieved by inhibiting Ang II-mediated harmful effects (25, 26, 47, 56, 106). Due to its anti-fibrotic and anti-inflammatory properties, ARBs effectively block Ang II-mediated AT1R activation and reduce acute lung injury in patients diagnosed with pneumonia, sepsis, and influenza (107–109). Therefore, ARBs blocking Ang II/AT1R pathway could overcome the adverse effects of ACE2 downregulation by SARS-CoV-2. On the other hand, several studies reported that ARBs beneficial effects could be correlated to the increase of ACE2 expression and activity in patients. Despite the beneficial properties of ACE2/Ang (1-7) axis (96, 110, 111), ACE2 upregulation after ARB treatment may open the door for viral entry, thus increasing the susceptibility of patients to SARS-CoV-2 infection.

Since the treatment of CVD patients is based on ARBs, their discontinuation in COVID-19 patients has been proposed to reduce ACE2 expression and, thus, the risk of a more severe infection associated with the increased viral entry. In fact, interruption of treatment may be more harmful than protective. For instance, discontinuing RAS inhibitors, including ARBs, in patients with an unstable clinical state (hypertension, heart failure, myocardial infarction), may result in a decline in clinical status and higher risks of mortality (60, 95). In fact, ARBs are reported to block Ang II/AT1R axis and to reduce acute lung injury pathogenesis. Interruption of ARBs might increase lung injury since Ang II-mediated AT1R activation is associated with vasoconstriction, oxidative stress, increased fibroproliferative, and inflammatory responses as well as lung oedema (54, 60, 112, 113). Importantly, studies have shown that these drugs can be protective against lung injury in SARS-CoV-1 patients by enhancing the protective arm of RAS (114). Also, retrospective studies showed that patients using ACEIs and ARBs present a lower risk of mortality and develop less severe cases as compared with those using other hypertensive drugs. On the other hand, these treatment regimens do not increase the risk of SARS-CoV-2 infection nor disease severity, as summarized in Table 1.

**Table 1:** The effect of ACEIs and ARBs on COVID-19 patients with CVD.

**Study design**	**Patient population**	**Outcomes**	**Institutes**	**References**
Multicenter, retrospective study	*N =* 476 COVID-19 patients with moderate (*n =* 352), severe (*n =* 54), or critical cases (*n =* 70) HT grup (*n =* 113) ACEIs/ARBs (51/113) Other drugs (62/113) Non-HT group (*n =* 363)	Higher incidence of comorbidities in the severe and critical groups as compared to the moderate group Patients receiving ACEIs or ARBs were more likely assigned to the moderate group than to the severe and critical groups	Three hospitals in Wuhan, Shanghai, and Anhui, China	(115)
Multicenter, retrospective study	*N =* 1128 COVID-19 patients with HT ACEI/ARB group (*n =* 188) Other drugs group (*n =* 940)	The use of ACEIs and ARBs in COVID-19 patients with HT is associated with a lower risk of all-cause mortality	Nine hospitals in Hubei, China	(116)
Multicenter, retrospective study	*N =* 609 COVID-19 patients HT group (*n =* 311) No treatment (60/311) ARB (76/311) ACEI (99/311) Other drugs (76/311) Non-HT group (*n =* 298)	Overall in-hospital mortality was 29% 42% among HT patients died in-hospital, after a median of 6 days from admission Patients receiving anti-HT drugs other than RASIs had a higher CCI, with a higher prevalence of COPD and CV comorbidities	Ten Italian hospitals	(117)
Single-center, retrospective study	*N =* 417 COVID-19 patients HT group (*n =* 51) No treatment (9/51) ACEI/ARB (17/51) Other drugs (25/51) Non-HT group (*n =* 366)	RASIs improve the clinical outcomes of COVID-19 patients with HT HT patients could benefit from the persistent or preferential usage of ACEIs and ARBs	Shenzhen Third People's Hospital, Shenzhen, China	(118)
Single-center, retrospective study	*N =* 251 COVID-19 patients HT group (*n =* 126) ACEI/ARB (43/126) Other drugs (83/126) Non-HT group (*n =* 125)	ARB/ACEI group had significantly lower concentrations of CRP and PCT ARB/ACEI group had a lower non-significant proportion of critical patients, and death rate This study supports the use of ARBs/ACEIs in COVID-19 patients with preexisting HT	Hubei Provincial Hospital of Traditional Chinese Medicine (HPHTCM) in Wuhan, China	(119)
Single-center, retrospective study	*N =* 1,8472 patients COVID-19 positive group (*n =* 1735) ACEI (116/1735) ARB (98/1735) COVID-19 negative group (*n =* 16,737) ACEI (1206/16737) ARB (884/16737)	This study supports various guidelines to continue current ACEIs or ARBs treatments during the COVID-19 pandemic This study found no association between ACEIs or ARBs use and COVID-19 test positivity	Cleveland Clinic Health System in Ohio and Florida, USA	(120)
Single-center, retrospective study	*N =* 1,178 COVID-19 patients HT group (*n =* 362) ACEI/ARB (115/362) Non-HT group (*n =* 816)	ACEIs/ARBs are not associated with COVID-19 severity or increased mortality rates This study supports current guidelines and societal recommendations for treating HT during the COVID-19 pandemic	The Central Hospital of Wuhan, China	(121)
Population based case-control study	*N =* 3,7031 patients COVID-19 positive group (*n =* 6,272) ARBs (1394/6272) ACEI (1502/6272) COVID-19 negative group (*n =* 30,759) ARBs (5910/30759) ACEI (6569/30759)	The use of ACEIs and ARBs was more frequent among patients with COVID-19 than among controls No evidence that ACEIs or ARBs affected the risk of COVID-19	Lombardy region, Italy	(122)
Single-center, retrospective study	*N =* 12,594 patients COVID-19 positive group (*n =* 5,894) HT (2573/5894) COVID-19 negative group (*n =* 6,700) HT (1784/6700)	No substantial increase in the likelihood of a positive test for COVID-19 or in the risk of severe COVID-19 among patients who tested positive in association with five common classes of anti-HT medications including ACEIs and ARBs	New York University (NYU) Langone Health system, New York, USA	(123)

*This table summarizes different retrospective studies evaluating the effect of ACEIs and ARBs on the risk of SARS-CoV-2 infection and disease severity in patients with preexisting HT. Altogether, the results showed that ACEIs and ARBs do not appear to be associated with a higher risk of SARS-CoV-2 infection, neither with disease severity and mortality. This evidence supports the current guidelines that discourage the discontinuation of ACEIs and ARBs in CVD patients infected with SARS-CoV-2. Of note, patients using ACEIs and ARBs are more likely to develop less severe symptoms and show improved clinical outcomes, reduced concentration of inflammatory markers as well as a lower risk of mortality compared to those using other antihypertensive drugs. In this context, recommendations are addressed toward the preferential use of RASIs for the management of hypertension in COVID-19 patients, all along with standard anti-viral medication. ACEI: angiotensin converting enzyme inhibitors, ARB, angiotensin receptor type 1 blockers; CCI, Charlson comorbidity index; COPD, chronic obstructive pulmonary disease; COVID-19, coronavirus disease-2019; CRP, C-reactive protein; CV, cardiovascular; CVD, cardiovascular disease; HT, hypertension; PCT, pro-calcitonin; RASI, renin-angiotensin system inhibitors; SARS-CoV-2, severe acute respiratory syndrome coronavirus 2*.

Taken together, more evidence is needed to support the proper use of ARBs for the treatment of COVID-19 and to exclude the risk of an increased viral entry. ARBs play a protective role in CVD patients by reducing the harmful effects of Ang II/AT1R axis while enhancing ACE2/Ang (1-7) protective axis. Therefore, ARB withdrawal can be potentially harmful rather than protective.

## Combined Therapy of Anti-COVID-19 Antibodies and Ang (1-7) Agonist for the Treatment of COVID-19 Patients

Several therapies are being investigated for the treatment of COVID-19 (124). Passive immunotherapy has also been reported as a treatment option to reduce mortality in many infectious viral diseases, including SARS-CoV-1 and severe influenza-related pathologies (125). Transfusion of anti-COVID-19 antibodies from recovered patients appears to be promising in severe patients. Recent studies showed that transfusion of convalescent plasma (CP) containing anti-COVID-19 neutralizing antibodies to COVID-19 critically ill patients, along with the conventional antiviral treatment, is associated with improvement in fever, inflammatory markers, lymphocyte count, viral clearance, and CT findings (126–128). In fact, Shen et al. conducted the first study describing the use of CP in COVID-19 patients. Indeed, 5 ARDS critically ill patients received CP from recovered healthy donors along with antiviral agents and methylprednisolone. All patients showed improvement of inflammatory markers, and the viral load became negative 12 days post transfusion (126). In another trial performed by Ye et al., 5 of 6 COVID-19 patients treated with CP demonstrated decreased pulmonary lesions based on their CT scan (129). Similar findings were reported by two other studies, revealing an overall improvement in clinical outcomes with no single death recorded during the treatment procedure (128, 130). Noteworthy, all the preliminary studies mentioned in Table 2 did not have control groups receiving CP alone, and the sample size of patients was generally limited in number. However, these studies established the safety and efficacy of anti-COVID-19 antibodies transfusion in critically ill patients. Therefore, CP was finally approved last August by the FDA as an investigational new drug for patients with life-threatening SARS-CoV-2 infection (144). On this basis, an FDA-initiated study on a cohort of 20,000 COVID-19 patients confirmed the safety of CP with low incidence of adverse events associated with transfusion (142). Recently, CP infusion was recommended during early stages of infection by Zeng et al. In this trial, 6 COVID-19 patients with respiratory failure received standard care along with CP treatment at a median of 21.5 days post-infection. Eventually, all patients tested negative for SARS-CoV-2 at 3 days post-infusion; however, 5 out of these 6 patients died, suggesting that CP is ineffective in reducing mortality in end-stage COVID-19 patients and should be initiated earlier (140). These findings were confirmed by Salazar et al., who reported a higher reduction in mortality rate in patients receiving CP transfusion within 44 hours of their hospitalization (143). These observations might be due to the late clinical deterioration observed in COVID-19 patients, related to hyper-immune attacks and inflammatory reactions, rather than a direct viral-effect since the peak of viral load is observed during the first week of infection (131). All clinical trials using CP for patients infected with SARS-CoV-1 or SARS-CoV-2 are presented in Table 2.

**Table 2:** The effect of convalescent plasma-based therapy on SARS and COVID-19 patients.

**Infectious agent**	**Study design**	**Patient population**	**Outcomes**	**Institutes**	**References**
SARS-CoV-1	Case series	*N =* 80 SARS patients with radiographic progression and hypoxemia Group 1: patients given CP before day 14 of illness (*n =* 48) Group 2: patients given CP after 14 days of illness (*n =* 32) No control group	A higher day 22 discharge rate was observed among patients who were given CP before day 14 of illness (58.3 vs. 15.6%) Overall mortality rate among 80 patients was 12.5%	Prince of Wales Hospital, Hong Kong, China	(131)
SARS-CoV-1	Single-center, retrospective non-randomized study	*N =* 40 SARS patients having clinical and radiographic deterioration despite methylprednisolone treatment Intervention group: patients given 3 doses of methylprednisolone steroids with CP (*n =* 19) Control group: patients given 4 or more doses of methylprednisolone (*n =* 21)	Discharge rate in intervention group was 73.4 vs. 19% in control group Mortality rate in intervention group was 0 vs. 23.8% in control group	Prince of Wales Hospital, Hong Kong, China	(132)
SARS-CoV-1	Case series	*N =* 40 SARS patients with pneumonia or ARDS Intervention group: patients receiving CP (*n =* NS) Control group: patients receiving other types of therapy (*n =* NS)	No mortality cases are reported in the intervention group Three mortality cases are reported in the control group	Medical College of Hong Kong Chinese University, China	(133)
SARS-CoV-1	Case series	*N =* 8 SARS patients Intervention group: patients receiving CP with antivirals and steroids (*n =* 3) Control group: patients receiving only antivirals and steroids treatments (*n =* 5)	Intervention group presents improved serial chest radiographs and decreased body temperature Viral load was no longer detectable after 24h of CP treatment	Taipei Municipal Hoping Hospital (TMHH), Taiwan	(134)
SARS-CoV-1	Cases report	*N =* 29 SARS patients Intervention group: 74 years old patient receiving CP after antivirals and steroids treatments (*n =* 1) Control group: younger SARS patients receiving antivirals and steroids treatments only (*n =* 28)	The patient receiving CP recovered from SARS within 21 days and have a shorter disease course than the control group	Beijing hospital, China	(135)
SARS-CoV-1	Case report	*N =* 1 SARS patient Intervention group: 57 years old patient receiving CP, antivirals and steroids treatments (*n =* 1) No control group	Patient showed improved Chest X-ray and decreased body temperature following CP treatment The patient made uneventful recovery	Prince of Wales Hospital, China	(136)
SARS-CoV-2	Cases report	*N =* 5 COVID-19 patients with ARDS, severe rapid progression pneumonia and continuously high viral load despite antiviral treatment Intervention group: patients receiving CP (*n =* 5) No control group	Body temperature normalized within 3 days, viral load became negative after 12 days and ARDS resolved in 4 patients out of 5	Shenzhen Third People's Hospital in Shenzhen, China	(126)
SARS-CoV-2	Multicenter, randomized clinical trial	*N =* 103 COVID-19 patients with severe ARDS, hypoxemia or life-threatening organ failure Intervention group: patients receiving CP in addition to standard treatment (*n =* 52) Control group: patients receiving standard treatment alone (*n =* 51)	51.9% of the intervention group showed clinical improvement (defined as patients discharged alive, or reduction in disease severity) vs. 43.1% in the control group Two patients from the intervention group experienced adverse events within hours after transfusion	Seven medical centers in Wuhan, China	(137)
SARS-CoV-2	Cases report	*N =* 2 COVID-19 patients with severe pneumonia and ARDS Intervention group: patients receiving antivirals and steroids treatments with CP (*n =* 2) No control group	The patients showed improved oxygenation and chest X-rays with decreased inflammatory markers and viral loads after CP infusion	Yonsei University College of Medicine, Seoul, Korea	(127)
SARS-CoV-2	Cases report	*N =* 10 COVID-19 patients with severe symptoms, respiratory distress, or hypoxemia Intervention group: patients receiving CP in addition to antiviral therapy and supportive care (*n =* 10) No control group	All patients achieved negative viral load, accompanied with an increase of oxygen saturation, improvement of liver function and alleviation of the overreaction of the immune system after plasma transfusion	Three participating hospitals in Wuhan, China	(128)
SARS-CoV-2	Cases report	*N =* 6 Critically ill COVID-19 patients with deteriorated symptoms after standard treatment Intervention group: patients receiving CP with anti-viral drug arbitol (*n =* 6) No control group	No adverse reactions were observed after plasma infusion After CP transfusion 5 of 6 patients showed improvement in the CT scan results All patients were discharged after achieving negative viral load	Wuhan Huoshenshan Hospital, Wuhan, China	(129)
SARS-CoV-2	Cases report	*N =* 4 COVID-19 patients with severe symptoms including ARDS Intervention group: patients receiving anti-viral and interferon-based drug in addition to CP (*n =* 4). No control group	All 4 patients achieved negative RT-PCR test results after 3-22 days of transfusion 2 of the 4 patients produced anti-SARS-CoV-2 IgG 14 days after CP transfusion	Dongguan Ninth People's Hospital and Xiangtan Central Hospital, China	(130)
SARS-CoV-2	Single-arm, multicenter trial	*N =* 46 COVID-19 patients with severe symptoms, including ARDS, low oxygen saturation levels and markedly elevated laboratory bio- markers Intervention group 1: one unit of CP in addition to standards treatment (*n =* 24) Intervention group 2: two units of CP in addition to standard treatment (*n =* 21) Intervention group 3: three units of CP in addition to standard treatment (*n =* 1) No control group	3 patients with important comorbidities died within 7 days of plasma transfusion 23% of survivors showed a clear chest X-ray, and 100% of them had improvement in laboratory biomarkers (C-reactive protein, ferritin and lactate dehydrogenase levels all decreased) after 7 days of transfusion All 43 patients achieved negative viral-load at the end of the study	Two university hospitals and one general hospital in northern Italy	(138)
SARS-CoV-2	Retrospective cohort study	*N =* 80 Critically ill Covid-19 patients with severe symptoms Intervention group: patients receiving CP along with standard care (including hydroxychloroquine, azithromycin, and lopinavir–ritonavir) (*n =* 40). Control group: patients receiving standard care only (*n =* 40)	Improvement in respiratory status achieved in 77.5% of intervention group vs 65% of control group Viral clearance achieved after 28 days of CP infusion in 65% of intervention group vs 55% in the control group Overall survival was 65% with no difference between the two groups	Hamad Medical Corporation (HMC), Qatar	(139)
SARS-CoV-2	Cases report	*N =* 21 COVID-19 patients with respiratory failure and required intensive care unit admission Intervention group: critically ill patients receiving CP transfusion at a median of 21.5 days' post infection in addition to standards treatment (*n =* 6) Control group: patients receiving standards treatment only (*n =* 15)	All the patients in the intervention group tested negative for SARS-CoV-2 RNA by 3 days after infusion 5 out of 6 patients in the intervention group died eventually	First Affiliated Hospital of Zhengzhou University, China	(140)
SARS-CoV-2	Cases report	*N =* 25 COVID-19 patients with severe and/or life-threatening disease Intervention group: patients receiving anti-inflammatory treatment (including tocilizumab and steroids) and anti-viral drugs, in addition to CP transfusion at median of 10 days' post symptom onset (*n =* 25) No control group	36% of patients had improvement in the clinical markers after 7 days of transfusion 76% of patients improved from baseline or been discharged after 14 days of transfusion Only one patient from the 25 died from a condition not caused by plasma transfusion	Houston Methodist hospitals, USA	(141)
SARS-CoV-2	Multicenter, single arm trial	*N =* 20 000 COVID-19 patients with severe life-threatening disease Intervention group: patients receiving CP in addition to standard care (*n =* 20,000) No control group	Overall 7 days' mortality rate was 12.96% The incidence of all serious adverse events related to plasma transfusion are low	FDA-initiated trial including multicentral /national hospitals	(142)
SARS-CoV-2	Multicenter, retrospective, non-randomized, propensity score-matched study	*N =* 5297 COVID-19 patients Intervention group 1: patients receiving CP with an anti-RBD IgG titer ≥1:1350 (*n =* 321) Intervention group 2: patients receiving CP with an anti-RBD IgG titer >1:150 but <1/1350 (*n =* 24) Intervention group 3: patients receiving CP with an anti-RBD IgG titer <1:150 (*n =* 6) Control group: propensity score-matched controls receiving standard treatment (*n =* 594)	Mortality was significantly decreased in patients who received plasma with an anti-RBD IgG titer of ≥1:1350 within 72 hours of admission 44 hours after hospitalization is optimal for transfusing COVID-19 patients with high-titer CP in order to prevent mortality 0,6% of patients developed significant adverse events related to plasma transfusion, including allergic reactions	Eight Houston Methodist hospitals, USA	(143)

*This table summarizes the majority of clinical trials using CP from recovered patients to treat critically ill patients infected with SARS-CoV-1 or SARS-CoV-2. As reported by several research groups, it seems that CP based therapy is generally associated with a higher discharge rate and a lower mortality risk. Also, this treatment plan is showed to be linked to improved clinical outcomes (body temperature, chest X-ray, oxygen saturation), decreased viral load, and a faster recovery. Growing evidence supports early administration of a high titer anti-COVID-19 antibodies within 72 h post-hospitalization. Analyses regarding the efficacy of CP as a standalone treatment strategy might be limited by the lack of control groups in some human trials and by the co-administration of standard care drugs (anti-inflammatory and anti-viral formulations) together with CP. Even though attention was directed toward the incidence of serious adverse events that can be related to plasma transfusion, it was FDA approved that the risk is low. ARDS, acute respiratory distress syndrome; COVID-19, coronavirus disease-2019; CP, convalescent plasma; NS, non-specified; RBD, receptor binding domain; SARS, severe acute respiratory syndrome; SARS-CoV-1, severe acute respiratory syndrome coronavirus 1; SARS-CoV-2, severe acute respiratory syndrome coronavirus 2*.

On the other hand, it is tempting to propose the use of Ang (1-7) agonists to overcome the harmful effects of SARS-CoV-2 infection in patients. Ang (1-7) has been reported to oppose the harmful effects of Ang II/AT1R axis by binding to MasR or to AT2R. In addition, it was shown to be cardiopulmonary protective through its anti-hypertensive, anti-thrombotic, anti-arrhythmic, and vasodilatory effects (145–147). Moreover, animal studies demonstrated that ARDS is associated with low Ang (1-7) levels and that Ang (1-7) upregulation reduces reactive oxygen species production and inhibits pulmonary fibrosis to control tissue damage. Besides, data showed that Ang (1-7)/MasR axis exhibits anti-inflammatory effects by inhibiting the NF-kB pathway and by reducing the production of pro-inflammatory cytokines such as TNF-α and IL-6 (53, 113, 148–151). In this context, Ang (1-7) oral formulation was also proved to attenuate the rupture of alveolar walls and behavioral changes in a mice model of elastase-induced emphysema (152).

The pathogenesis of SARS-CoV-2 infection is mediated through over-activation of the inflammatory response and an increased cytokine production (113, 148, 149). This could be related to the inhibition of the anti-inflammatory ACE2/Ang (1-7)/MasR axis by binding of the virus. Therefore, the severity of COVID-19 could be attenuated by Ang (1-7) administration, which may restore the anti-inflammatory response via MasR activation (153). Several Ang (1-7) agonists are available such as AVE-0991 (154), hydroxypropyl β-cyclodextrin (HPβCD)/Ang (1-7) (155, 156), cyclic angiotensin (1-7) (157), CGEN-856, and CGEN-857 (158). In animal models, these agonists exert their protective effects, such as vasodilation and improved cardiac remodeling, by binding to MasR, thus mimicking Ang (1-7) effects with high *in vivo* stability (158, 159). Unfortunately, not all Ang (1-7) agonists have been evaluated in human subjects; thus, the safety data is lacking for some of these drugs. Importantly, FDA has granted a pharmaceutical formulation of Ang (1-7), called TXA127, an orphan drug for the treatment of several conditions, including Duchenne muscular dystrophy and pulmonary arterial hypertension (160). Several clinical trials on TXA127 were announced since 2008, some of which were terminated or withdrawn for unknown reasons. For instance, phase I/II clinical trials conducted on cancer patients showed that Ang (1-7) drug is safe, well-tolerated, with no mortality rate and low-grade adverse events such as fatigue, injection site reaction, and flu-like symptoms (161, 162). Interestingly, two double-blind, placebo-control, randomized clinical trials are currently being conducted in Brazil (NCT04633772), Israel (NCT04605887), and New York (NCT04401423) on patients with severe COVID-19 cases. The purpose of these studies is to test the safety and efficacy of TXA127 and to determine whether Ang (1-7) infusions prevent respiratory failure, acute kidney injury, and multi-organ damage due to the management of inflammation. Considering the increasing spread and number of deaths due to COVID-19, it is extremely urgent to evaluate TXA127 and other Ang (1-7) agonists as possible treatments for seriously ill patients.

In the light of the vital role of Ang (1-7) in lung protection and the promising results of anti-COVID-19 antibodies based therapy, combination treatment of Ang (1-7) agonists along with anti-COVID-19 antibodies may be the ideal therapeutic intervention to alleviate COVID-19 severity (Figure 3).

## Conclusion and Limitations

This review summarizes the treatment strategies targeting ACE2 viral receptor, either directly or indirectly, in the context of COVID-19. Combination therapy using Ang (1-7) and CP in patients infected with SARS-CoV-2 appear to be the most promising alternative. Although the clinical potential of Ang (1-7) agonists has been evidenced in numerous human trials (161, 162); however, its use is limited due to the absence of studies validating its safety and efficacy in COVID-19 patients. In addition, a stable oral Ang (1-7) compound covering a broad range of patients is still lacking (163). Importantly, prolonged exposure to other immuno-suppressor drugs such as corticosteroids might increase the occurrence of secondary infections; thus, evaluating the risk of opportunistic infections in patients treated with Ang (1-7) appear to be of great value (164, 165). Furthermore, steroids possess salt retention activities that might increase the stress over the cardiovascular system.

On the other hand, CP therapy may encounter several challenges that should be taken into consideration despite its proven benefits. One such limitation is its availability; in fact, a shortage in the number of plasma donors could be seen in situations of rapid disease spread resulting in an increased number of infected patients compared to those that have recovered. This would be especially significant and serious for the rare blood group patients. In addition, potential risks can be directly associated with CP regimen. These risks include, among others, the transmission of harmful pathogens such as HIV, hepatitis B/C, and syphilis. Moreover, other transfusion-related events may occur following CP treatment, including allergic, anaphylactic, or hemolytic reactions, fever, and transfusion circulatory overload (166). Furthermore, transfusion can be associated with transfusion-related acute lung injury (167). Although this complication is not common; however, this possibility should not be ignored since COVID-19 patients are at high risk of developing pulmonary disease (168). Importantly, all studies on CP transfusion as a COVID-19 treatment showed that these severe adverse reactions are infrequent.

The presence of a confounding variable in most CP studies makes it difficult to prove the efficacy of CP transfusion as a stand-alone treatment because patients are mostly receiving a standard care treatment such as anti-viral and anti-inflammatory drugs along with anti-COVID-19 antibodies (128, 141). In addition, the safety and efficiency of CP in pregnant women and pediatric patients have not been evaluated yet. Noteworthy, several ongoing studies are covering both population groups (169). Finally, since the immune-competent population contributed to the generation of new viral strains in South Africa and the United Kingdom, new adaptations of the virus raise the concern about the possibility of escaping viral neutralization by convalescent antibodies. In this context, a very recent study reported that SARS-CoV-2 has the ability to generate new mutations in its viral spike, which is typically recognized by antibodies, thus facilitating the escape from neutralization (170).

Taken together, more clinical trials are warranted to prove the safety and efficacy as well as the synergistic therapeutic effects of this combination treatment procedure.

## Author Contributions

AN and KZ proposed the hypothesis and originated this work. All authors listed have made a substantial, direct and intellectual contribution to the work, and approved it for publication.

## Conflict of Interest

The authors declare that the research was conducted in the absence of any commercial or financial relationships that could be construed as a potential conflict of interest.

## Abbreviations

α7-nAChR: α7 subtype of the nicotine acetylcholine receptor
ARDS: Acute respiratory distress syndrome
Ang I: angiotensin I
Ang (1-7): angiotensin (1-7)
Ang (1-9): angiotensin (1-9)
Ang II: angiotensin II
ACE: angiotensin converting enzyme
ACE2: angiotensin converting enzyme 2
ACEIs: angiotensin converting enzyme inhibitors
AT1R: angiotensin type 1 receptor
AT2R: angiotensin type 2 receptor
ARBs: angiotensin type 1 receptor blockers
Abs: antibodies
CV: cardiovascular
CVD: cardiovascular disease
CFR: case fatality rate
CCI: charlson comorbidity index
COPD: chronic obstructive pulmonary disease
CP: convalescent plasma
COVID-19: coronavirus disease-2019
CRP: C-reactive protein
DPP4: dipeptidyl peptidase 4
FP: fusion peptide
MasR: G-coupled receptor Mas
GPCR: G protein-coupled receptors
HR1: heptad repeat region 1
HR2: heptad repeat region 2
HT: hypertension
MERS-CoV: Middle East respiratory syndrome coronavirus
NS: non-specified
PCT: pro-calcitonin
RBD: receptor-binding domain
rhACE2: recombinant human ACE2
RAS: renin angiotensin system
RASIs: renin angiotensin system inhibitors
SARS-CoV-1: severe acute respiratory syndrome coronavirus
SARS-CoV-2: severe acute respiratory syndrome coronavirus 2
S protein: spike protein
S1: S protein subunit 1
S2: S protein subunit 2
TMPRSS2: transmembrane protease serine 2.
